# *In vivo* characterization of hair and skin derived carbon quantum dots with high quantum yield as long-term bioprobes in zebrafish

**DOI:** 10.1038/srep37860

**Published:** 2016-11-25

**Authors:** Jing-Hui Zhang, Aping Niu, Jing Li, Jian-Wei Fu, Qun Xu, De-Sheng Pei

**Affiliations:** 1 College of Materials Science and Engineering, Zhengzhou University, Zhengzhou 450052, China; 2 Chongqing Institute of Green and Intelligent Technology, Chinese Academy of Sciences, Chongqing 400714, China

## Abstract

Carbon quantum dots (CDs) were widely investigated because of their tunable fluorescence properties and low toxicity. However, so far there have been no reports on *in vivo* functional studies of hair and skin derived CDs. Here, hair derived CDs (HCDs) and skin derived CDs (SCDs) were produced by using human hair and pig skin as precursors. The quantum yields (QYs) of HCDs and SCDs were quite high, compared to citric acid derived CDs (CCDs). HCDs and SCDs possess optimal photostability, hypotoxicity and biocompatibility in zebrafish, indicating that HCDs and SCDs possess the capacity of being used as fluorescence probes for *in vivo* biological imaging. The long-time observation for fluorescence alternation of CDs in zebrafish and the quenching assay of CDs by ATP, NADH and Fe^3+^ ions demonstrated that the decaying process of CDs *in vivo* might be induced by the synergistic effect of the metabolism process. All results indicated that large batches and high QYs of CDs can be acquired by employing natural and nontoxic hair and skin as precursors. To our knowledge, this is the first time to report SCDs, *in vivo* comparative studies of HCDs, SCDs and CCDs as bioprobes, and explore their mechanism of photostability in zebrafish.

Carbon quantum dots (CDs), the novel quasi-spherical nanoparticles with size less than 10 nm, have caught considerable attention because of their fascinating advantages, such as hypotoxicity, photochemical stability, hydrophilicity, particular electronic properties and the unique wavelength-dependent photoluminescence properties^1^. Hence, they have multiple applications in the research fields of photocatalytic energy conversion^2^, catalysis^3^, fluorescence labeling of cells^4^, food detection^5^, photovoltaic conversion^6^, and multifunctional sensor^7^. Compared to organic dyes and semiconductor QDs, CDs are biocompatibility and noninvasive for cells and other living organisms at much higher concentration levels, with the primary elements of carbon, oxygen, hydrogen, and nitrogen. Furthermore, due to the existence of hydroxyl, carboxyl or amino groups, the surface of CDs can be readily modified with different functional groups, which enables CDs more prospects for tuning their physicochemical properties. Therefore, the CDs possess the potential as desirable fluorescent probes to visualize biological systems both *in vivo* and in *vitro*^8^,^9^.

Recently, diverse methods of preparing CDs, such as chemical oxidation^10^, electrochemical carbonization^11^, laser passivation^12^, microwave irradiation^8^,^13^, and hydrothermal/solvothermal treatment^14^,^15^, had been carried out. The raw materials used to prepare CDs include chitosan^16^, citric acid and ethylenediamine^17^, activated carbon^10^, glucose^14^, and poly (ethylene glycol)^18^. Even biomass banana juice^19^ was also used to prepare multiple CDs. Nevertheless, current synthetic methods may involve complicated processes, expensive equipments, hazardous precursors, uncontrollable nanostructure, and low quantum yield, which gravely restrict the large-scale production and applications of CDs as fluorescent probe. Hence, it is necessary to develop a simple, rapid, economical, and environmentally friendly synthetic method to generate CDs on a large scale. Previous reports showed that nitrogen doping is a powerful approach to tune the photoluminescence properties of CDs and enhances the fluorescence quantum yield of CDs by using some nitrogen-containing organic compounds or adding nitrogen sources^1^,^20^,^21^. To select the molecular precursor of CDs, the abundant and biocompatible protein-rich materials are the best candidates, because protein itself contains rich nitrogen and carbon elements, which can be modified by some functional groups to improve the affinity and fluorescence property. Among all reported preparation approaches, microware irradiation method is rapid, scalable, cost effective, and eco-friendly, but poor control over sizes. Interestingly, carbonization method is size and nanostructure controllable^1^. If these two methods can be combined together, it will facilitate to obtain CDs with high fluorescent quantum yields (QYs) and controllable lateral dimensions, which is confirmed in this study.

Ideally, the fluorescent bioprobes ought to possess excellent dispersibility, stability, hypotoxicity and good biocompatibility. In consideration of the inherent chemical composition and nanoscale properties, the hypotoxicity and biocompatibility of CDs in living organism depend on the concentration and superficial chemical components of CDs^22^,^23^. Therefore, it should be ensured the safety of CDs before applying them for bio-applications, such as bioluminescence imaging. In order to prove the feasibility of CDs used as fluorescent probes, several models have been used to examine the dispersibility, toxicity and biological compatibility of CDs both *in vitro* and *in vivo*, such as cells^4^,^24^,^25^, bacteria^26^,^27^, mouse^28^,^29^ and zebrafish^30^. Among all above models, zebrafish (*Danio Rerio*) is a favorite model for the studies on the practicability of CDs as bioprobes, due to its short generation time, low husbandry cost, facile genetic screening and expression, prompt embryonic development and transparent embryo for optical *in vivo* imaging. Most importantly, its genomes are strikingly homologous to human genes^31^. Up to now, zebrafish has been used as a model to study cardiovascular diseases^32^, drug screening and validation^33^, DNA damage and repair^34^ as well as the toxicity testing^35^,^36^,^37^. The fish at early developmental stages are very susceptive to toxic substances, so the embryo is more accurate and appropriate for assessing the toxicity, the transport and biocompatibility of xenobiotics^38^.

Although CDs have been extensively studied, the photoluminescence mechanism and *in vivo* longtime decay of CDs are still elusive. Here, hair derived CDs (HCDs) and skin derived CDs (SCDs) with high QYs were produced by using protein-rich human hair and pig skin as the precursors, and citric acid CDs (CCDs) produced by microware irradiation method were chosen as control to investigate the effects of precursors on photoluminescence properties. The microstructure, size and chemical composition of the CDs were characterized deeply and the QYs of three CDs were calculated. To demonstrate the feasibility of CDs as fluorescent probes, the effects of a series concentrations of CDs, the absorption, distribution, metabolism and excretion (ADME) of CDs were tested in zebrafish. Moreover, the quenching assays were performed to detect the decaying mechanism of CDs in zebrafish by adding ATP, NADH and Fe^3+^ ions to CDs solution. As far as we know, the SCDs from protein-rich pig skin were firstly reported here. *In vivo* comparative studies and mechanism studies on photostability in zebrafish were also firstly explored for three CDs (HCDs, SCD and CCDs) as bioprobes.

## Results

### Preparation and characterization of CDs

**Figure 1:**
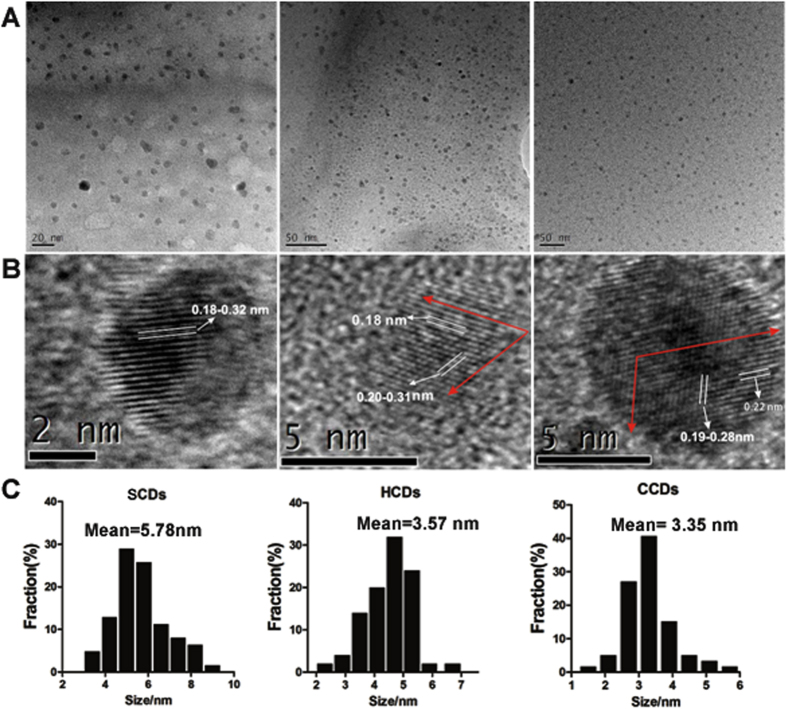
Characterization of CDs by using Transmission Electron Microscopy (TEM). (**A**) TEM images; (**B**) HRTEM images; (**C**) particle size distribution of SCDs, HCDs and CCDs.

The morphology, size and microstructure of the resultant CDs were characterized by transmission electron microscopy (TEM) (Fig. 1). Obviously, the microsphere morphology of three as-synthesized CDs was very small (Fig. 1A). The size distributions of the three CDs were mostly concentrated on the range of 2–6 nm with the average diameters of 5.78 nm (SCDs), 3.35 nm (CCDs) and 3.57 nm (HCDs), respectively (Fig. 1C). The structural details and the atomic lattice fringes of CDs could be seen distinctly in HR-TEM (Fig. 1B). It was shown that the CDs had lattice spacing of about 0.18–0.23 nm, among which the lattice spacing of 0.18 nm corresponded to the [102] facet of graphitic carbon^39^, and the lattice spacing of 0.23 nm was very close to the [100] facet of graphene^40^, which indicated that the CDs possessed a high-graphitizing structure. Powder X-ray diffraction analysis was conducted to investigate the crystalline structure of CDs.

**Figure 2:**
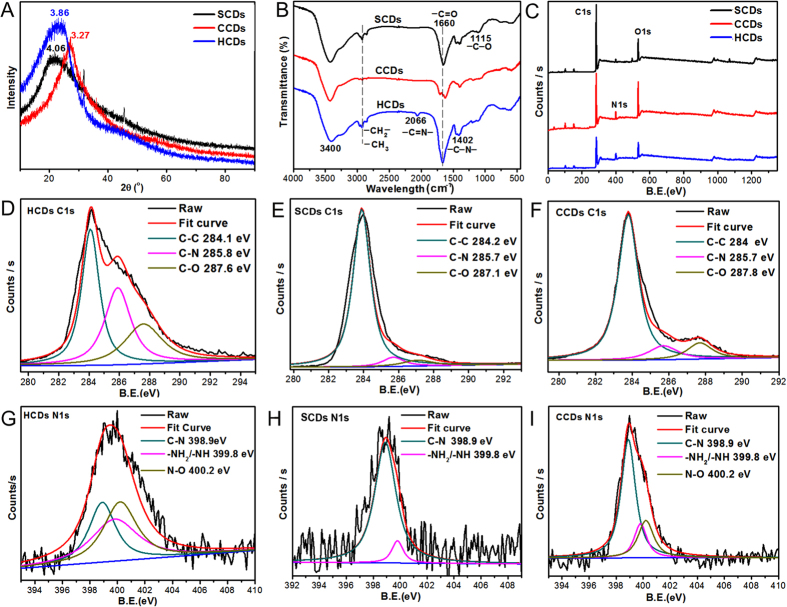
Characterization of chemical structure of CDs. (**A**) XRD patterns; (**B**) FT-IR (Fourier transform infrared) spectra; (**C**) XPS spectra of HCDs, SCDs and CCDs; (**D**) C1s profile of HCDs; (**E**) C1s profile of SCDs; (**F**) C1s profile of CCDs; (**G**) N1s profile of HCDs; (**H**) N1s profile of SCDs; (**I**) N1s profile of CCDs.

As shown in Fig. 2A, when the value of 2θ was 23.04 for HCDs or 21.87 for SCDs, the patterns of HCDs and SCDs possessed a broad (002) peak without sharp peaks, and the corresponding value of interlayer spacing was 3.86 Å for HCDs and 4.06 Å for SCDs, which indicated that a disordered amorphous carbon structure was formed. The value of interlayer spacing for HCDs and SCDs was higher than that of graphite (0.34 nm)^41^,^42^, indicating an increase in amorphous nature, which might be ascribed to the presence of functional groups at the surface of the CDs.

Meanwhile, the Fourier Transform Infrared (FTIR) spectroscopy was recorded to identify the organic functional groups on CDs. As depicted in Fig. 2B, the as-prepared CDs mainly contained C=O (carboxyl/carbonyl, stretching at ~1660 cm^−1^), —OH (hydroxyl, stretching at ~3400 cm^−1^), and C—N (peptide bond, stretching at 1402 cm^−1^). There were a weak peak at 2865 cm^−1^ and 2921 cm^−1^ for HCDs and SCDs, which were attributed to the stretching vibration of —CH_2_– and —CH_3_. Simultaneously, the weak C—O stretching band occurred at ~1115 cm^−1^ (alkoxy). Additionally, the weak peak existing at 2066 cm^−1^ for HCDs was related to —C=N— band, which might result from remained histidine content after microwave assisted carbonization. Noticeably, among these three CDs, the C=O stretching peak of HCDs was the strongest. The massive carbonyl as well as a small amount of amino and hydroxyl groups indicated the presence of carboxylic acid and amino/oxygen-containing functional groups on the surface of CDs.

To deeply detect the surface chemical composition of the three CDs, X-ray photoelectron spectroscopy (XPS) analysis was performed. As shown in the Fig. 2C, three dominant peaks at 284.8, 399.1 and 531.5 eV were ascribed to C1s, N1s and O1s, indicating that the surface of CDs were covered with C, N and O. To acquire the elements information about the binding feature on the surface, the C1s, N1s and O1s regions in the expanded XPS spectra were detected. The high-resolution spectrum of C1s indicated that the C1s curve fitting was optimized into three freestanding peaks: the graphitic carbon (C–C, 284.0–284.2 eV), N-sp^2^ C (C–N, 285.7–285.8 eV), and hydroxyl or carbonyl groups (C–O and C=O, 287.1–287.8 eV)^43^,^44^ for all CDs, while peaks of C-N and C-O/C=O in HCDs were stronger than that of SCDs and CCDs. Spectrum of N1s (Fig. 2G–I) could be resolved into three peaks for HCDs and CCDs at 398.9, 400.2 and 399.8 eV, as well as two peaks for SCDs with binding energies at about 398.9 and 399.8 eV. Those peaks indicated that C-N and N-H/NH_2_ were mostly formed by the nitrogen in the CDs, indicating that the CDs were successfully doped with nitrogen atoms. The major elemental composition of the CDs surface was shown in Table 1. All results indicated that carbon was the most plentiful constituent for all CDs. Significantly, the nitrogen content of HCDs was the highest of three CDs. 2.43–9.66% of nitrogen and 73.60–82.93% of carbon further confirmed that CDs were the nitrogen-doped and carbon-rich materials. The XPS results showed that the as-synthesized CDs might have functional groups like -COOH, -OH and –NH, which were well agreed with the FT-IR results.

**Figure 3:**
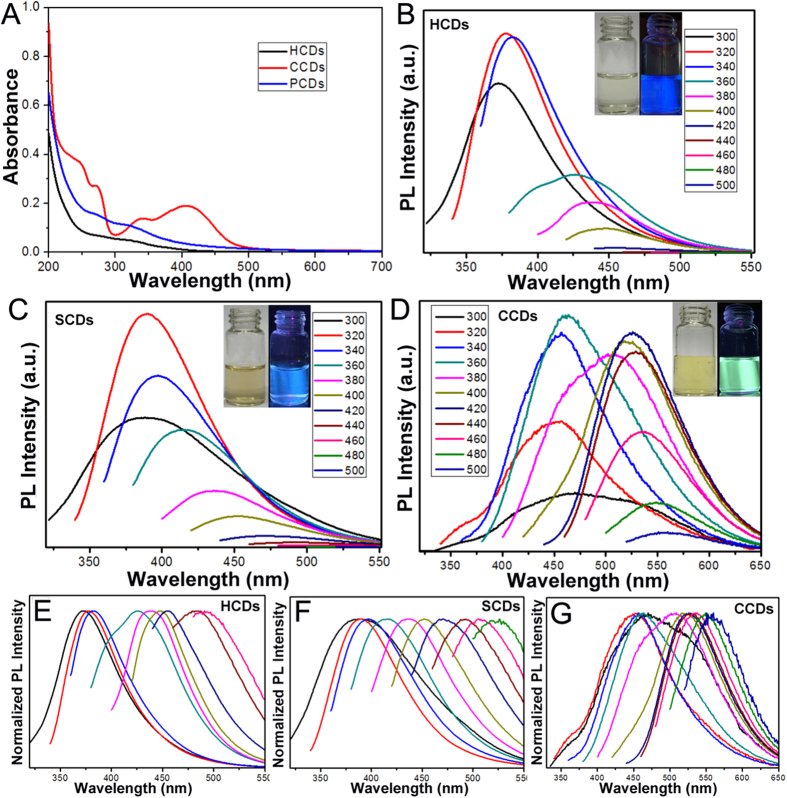
The photoluminescence characterization of CDs. (**A**) UV-vis absorption; (**B–D**) Fluorescent luminescence emission spectra of HCDs, SCDs and CCDs with progressively longer excitation wavelengths in 20 nm increments from 300 nm to 500 nm. Inset: the fluorescent images of CDs in deionized water solution under the UV light before and after the UV light on. (**E–G**) Normalized emission spectra of HCDs, SCDs and CCDs with the same excitation wavelengths.

To gain the optical properties of the CDs, the UV–vis absorption and photoluminescence emission spectra were performed. In the UV–vis spectra, the aqueous solution of HCDs and SCDs exhibited broad absorption spectrum without any obvious peak, while there were several absorption peaks focused on 270 nm, 340 nm and 405 nm in the aqueous solution of CCDs (Fig. 3A). Excitation-dependent luminescence was observed for three CDs as the increasing excitation wavelength from 300 nm to 500 nm, resulted in red-shifted of the emission spectrum from 450 (blue) to 550 nm (green) (Fig. 3B–D). The normalized emission spectra of CDs in Fig. 3E–G also clearly and intuitively illustrated that the emission of CDs were excitation-dependent. As the excitation wavelengths changed from 300 nm to 500 nm, the emission spectra was observed with the strongest emission around 380 nm under the excitation of 320 nm for HCDs and SCDs, as well as 450 nm strongest emission under the excitation of 360 nm for CCDs (Fig. 3B–D), which resulted in the different fluorescent colors of HCDs, SCDs and CCDs, *i.e.* strong blue luminescence for HCDs and SCDs as well as strong yellow green luminescence for CCDs under UV irradiation (The inset of Fig. 3B–D). The QYs of the as-prepared three CDs were characterized by quinine sulfate as standard, the results were 86.06%, 51.35%, and 19.73% for HCDs, SCDs and CCDs, respectively (Table S1). Obviously, the QYs of HCDs and SCDs were much higher than that of CCD, which indicated that protein-rich materials could be chosen as the optimal precursors for preparation of CDs, compared to other raw materials.

### Biocompatibility and fluorescence imaging of CDs in zebrafish

**Figure 4:**
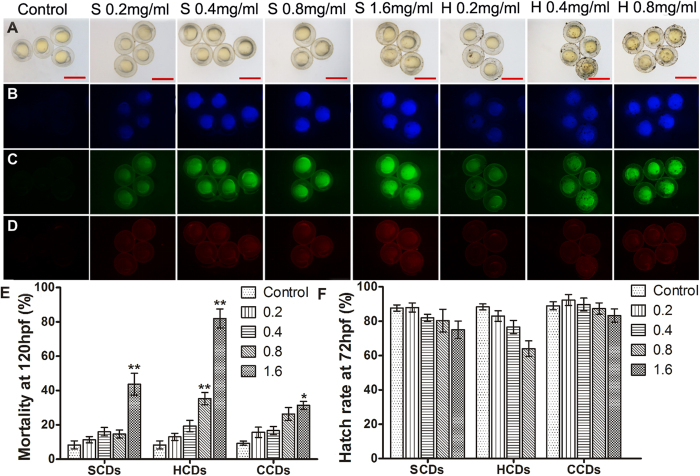
The fluorescent imaging of CDs in zebrafish and biocompatibility of CDs. (**A–D**) Fluorescent microscopic images of bright field and fluorescent field (B blue, C green, and D red) of zebrafish embryos at 24 hpf after exposure to different concentrations of SCDs and HCDs. Scale bars, 1000 μm. (**E,F**) Effects of exposure concentration of SCDs, HCDs and CCDs on zebrafish mortality at 120 hpf and hatch rate at 72 hpf (n = 50). Single asterisk (*) indicated significant difference compared to control at P < 0.05, and double asterisks (**) indicated significant difference, compared to control at P < 0.01. Values represented the mean ± standard error (SE) of three replicates.

The as-prepared CDs possessed outstanding photophysical and photochemical properties, such as high fluorescence QYs, which might enable them to be bioprobes. However, in view of the inherent chemical composition and nanoscale properties, the bioluminescence imaging of these CDs still needs to be exploit based on the dose limitation of different CDs. To survey the applicability of CDs as fluorescent biomarkers in practical biological environment, zebrafish were chosen as the model to evaluate the biocompatibility and biotoxicity of as-synthesized CDs. The developmental toxicity of CDs for zebrafish was examined by exposing embryos to the different concentrations of CDs for 20 h from 4 hpf. As shown in Fig. 4 and Fig. S1, the embryos incubated with CDs exhibited blue, green and red fluorescence under the ultraviolet (330–385 nm), blue (450–480 nm) and green (510–550 nm) light excitation, respectively. On the contrary, no fluorescent signals were detected in the control embryos without CDs labelling. Nevertheless, the red fluorescence was relatively weaker, compared with blue and green fluorescence. It was related to the fluorescent characterization of CDs, *i.e.* the emission spectra of CDs was concentrated in the green and blue fluorescent scope, which was consistent with the photoluminescence results. High magnification revealed that CDs were distributed over the whole embryos after 20 h exposure for SCDs and HCDs (Fig. 4A and Fig. S1A). The fluorescent intensity in the embryos increased with the enhancement of the concentration. SCDs and HCDs showed pretty photoluminescence imaging *in vivo* zebrafish, while for CCDs, the fluorescence primarily adhered on the surface of chorion and there was almost no obvious fluorescence detected in the embryos after exposure to different concentration of CCDs for 20 h, even when the exposure concentration reached up to 2 mg/ml (Fig. S1). By comparing with the bright field images of embryos (Fig. 4 and Fig. S1), the embryos incubated in all CDs solutions were developmentally normal. In addition, the mortality of embryos at 120 hpf was calculated. The results showed that mortality increased with the increasing concentration of CDs, and there was no apparent interference on embryos, when the concentrations were lower than 0.8 for HCDs and 1.6 mg/ml for SCDs and CCDs, respectively, which indicated the hypotoxicity and high biocompatibility of three CDs (Fig. 4E). For embryos soaked in different concentrations of HCDs and SCDs solutions, there were almost the same hatching rate at 72 hpf and no significant difference between three tested and control groups, demonstrating that CDs has almost no effect on developmental delay (Fig. 4F).

To investigate the long time hypotoxicity of HCDs and SCDs, the exposure time was prolonged to 44 h in 0.4 mg/ml CDs. As shown in Fig. S2, the embryos developed normally for HCDs and SCDs, but there was still no fluorescence seen in embryos for CCDs treatment, which indicated that HCDs and SCDs were low-toxic indeed and competent for *in vivo* imaging. However, some CDs particles were aggregated on the zebrafish chorion, and the aggregation of the CDs on the surface of the chorion were much more serious with the increasing concentration of HCDs and SCDs (Fig. 4A). While the CCDs were evenly distributed on the surface of chorion without any large particles seen (Fig. S1). When the exposure concentrations of CCDs were increased, zebrafish embryos became darker in the bright field, demonstrating that more CCDs were adhered on the chorion. It was assumed that a number of functional groups with same charges existed on the surface of CCDs prevented CCDs to enter zebrafish chorion. The above results indicated that HCDs and SCDs are more competent for *in vivo* imaging in zebrafish, compared with CCDs.

CCDs had been confirmed not to enter into embryos by chorion. To better compare the properties of CDs *in vivo*, the microinjection method was implemented to detect the affinity of different CDs to embryos. After three CDs solutions were individually injected into zebrafish embryos, the fluorescent images were taken in 30 min immediately. As shown in Fig. S3, the SCDs and HCDs formed a bright fluorescent spot but were not dispersal, while the CCDs dispersed into the whole embryo completely. This phenomenon showed that CCDs possessed high dispersibility in embryos and were non-specific for organs compared to HCDs and SCDs, which indicated that all three CDs could be introduced into embryos directly by microinjection strategy. Thus, fluorescence probes used for different purposes should consider not only its optical properties, but also chemical properties. To further verify the affinity and biocompatibility of CDs, the 10-day-old normal larvae were investigated to exclude the influence of chorion. The 10-day-old larvae were raised in 0.4 mg/ml CDs solutions for 48 h, and the result were shown in Fig. S4. Due to the 10-day-old larvae had developed well, the CDs was difficult to penetrate into the body. As shown in Fig. S4, all 10-day-old larvae exposed in CDs were survived and developed normally with fluorescence concentrating in the belly and gut. The interpretation was that the CDs probably entered the fish by ingestion, which further verified that the as-prepared CDs had low toxicity and high biocompatibility.

### Detection of photostability and decaying process of CDs *in vivo*

**Figure 5:**
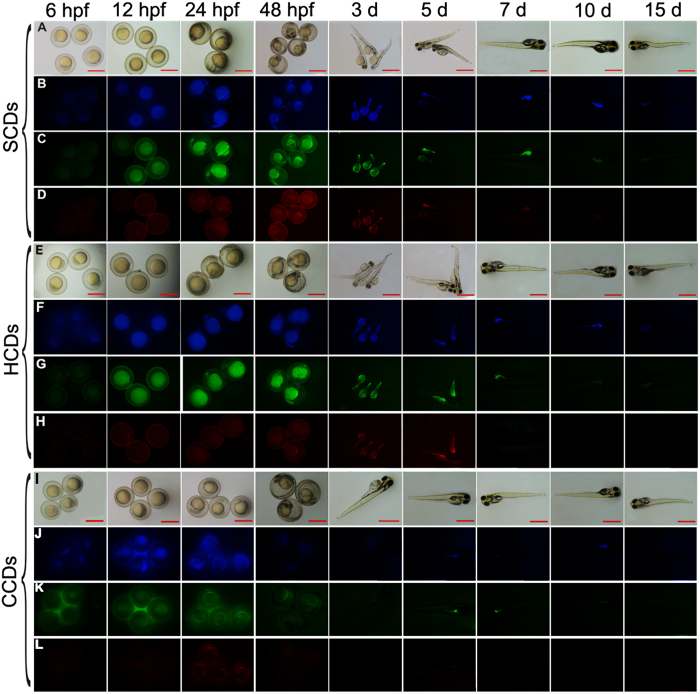
The photoluminescence decay of CDs in zebrafish. The fluorescent microscopic images of bright field and fluorescent field of zebrafish embryos after exposure to 0.4 mg/mL HCDs, SCDs and CCDs solutions for 2 days at different time points. Scale bars, 1,000 μm.

To test the photostability and decaying process of CDs as bio-probes in zebrafish, the spatio-temporal effect of CDs on zebrafish was detected after embryos were incubated for 44 h in 0.4 mg/ml CDs, which was a confirmed concentration as bio-probe. The fluorescence alteration and distribution in zebrafish was traced for 1 days. As shown in Fig. 5, during the 44 h incubation, the fluorescence intensity was extremely weak in the embryos at 6 hpf after incubated for 2 h in CDs solution, illustrating that it needed time for CDs to enter into the embryos through chorion and penetrate into yolk. After incubation with CDs for 8 h, the strong fluorescence was observed in embryos at 12 hpf. When the incubation time was lengthened, the fluorescence intensity remained unchanged during the period of incubation, demonstrating that CDs permeated into embryos and reached saturation after incubation for 8 h. What’s more, there was no developmental damage to embryos exposed to CDs for 44 h. After 72 hpf, the embryos were hatched into larvae with normal development and the fluorescence was still bright without further supplementary CDs. Nevertheless, the fluorescence intensity began to decrease at the 5th day and mostly concentrated on belly and tail of zebrafish larvae (Fig. 5 and Fig. S5). With the development of larvae, the fluorescence became increasingly weaker, and there was only little fluorescence in the ten-day old larvae. After 15 d culture of larvae, the fluorescence completely diminished. CDs might be removed from larvae by the digestive system (fluorescence in the belly and gut), or quenched by some inner-body metabolite or substance in zebrafish. The photostability and decaying process of CDs as bioprobes in zebrafish illustrated that the fluorescence imaging had a dose-dependent and time-dependent strategy. The results indicated that the decaying process in embryos was slow and the CDs were photostability *in vivo*. Moreover, the slow decaying process of CDs *in vivo* showed that the SCDs and HCDs were better as bioprobes for long-term observation of *in vivo* targets, compared to the CCDs.

### Fluorescence quenching mechanism of CDs in zebrafish

**Figure 6:**
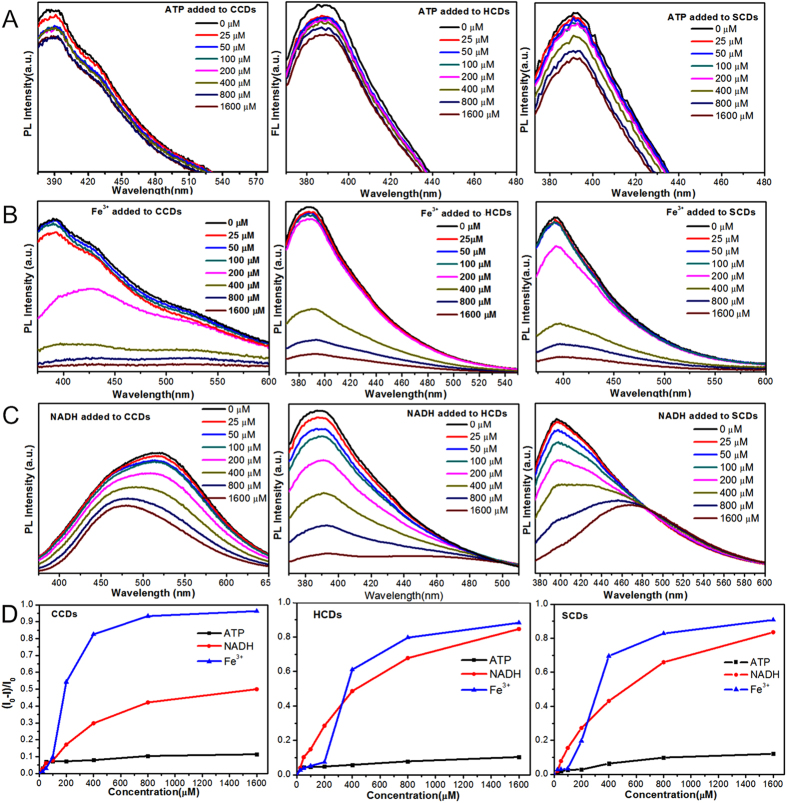
The quenching effects of ATP, NADH and Fe^3+^ ions on CDs. (**A–C**) The photoluminescence emission spectra of three CDs after added increasing concentrations (0–1,600 μM) of ATP, Fe^3+^ ions and NADH in PBS buffer. (**D**) Quenching efficiencies of ATP, Fe^3+^ ions and NADH on three CDs. I and I_0_ are the FL intensities of CDs at 340 nm excitation in the presence and absence of ATP, NADH and Fe^3+^ ions, respectively.

Previous reports showed that the phenolic hydroxyl and carboxyl groups of the CDs had specific coordination interaction with ATP, NADH and Fe^3+^ ions^15^,^17^,^45^, which hindered the radiative recombination of electrons and holes trapped on the CDs surface and caused the fluorescence quenching of CDs. To further investigate the decay mechanism of CDs *in vivo* and their potential practical applications, the influences of ATP, NADH and Fe^3+^ ions on CDs were detected. As shown in Fig. 6A–C, after added ATP, NADH and Fe^3+^ ions, the photoluminescence intensity of all CDs presented dose-dependent decrease with increasing concentrations of three additive. For three CDs, the quenching efficiency of three agents was Fe^3+^ ions > NADH > ATP orderly in high concentrations (200 μM–1,600 μM). It was noteworthy that there was another spectra occurring around 480 nm in the quenching process of NADH for CCDs and SCDs (Fig. 6C), which was corresponding with the peak of NADH spectra (Fig. S6), and demonstrated that excess NADH was added. There was no other peak seen for the quenching of HCDs, which might contribute to the high QYs of HCDs. However, in low concentrations (less than 200 μM), the quenching efficiency of NADH was higher than Fe^3+^ ions (Fig. 6D). As shown in Fig. S7, the fluorescent spectra and images of CDs quenched by Fe^3+^ ions were achieved at high concentrations with increasing concentration of Fe^3+^ ions, which obviously displayed the quenching process of Fe^3+^ ions. For low concentrations of NADH, as shown in Fig. S8, the linear calibration plot in the concentration range of 0–100 μM was simulated with the correlation coefficients of 0.97657 (HCDs), 0.98957 (SCDs) and 0.98543 (CCDs), respectively. It indicated that three CDs could be used as probes for quantifying NADH in low concentration. Meanwhile, the above results showed that three CDs could be quenched by three added substances. Numerous enzymes, ATP and other ions like Fe^3+^ ions could quench the fluorescence and cause the decay of the CDs in zebrafish embryo *in vivo*. Moreover, the as-synthesized CDs could also be used as sensitive probes for detecting of NADH, ATP and Fe^3+^ ions.

## Discussion

Although there have been some reports on CDs used as fluorescence probes^30^, the different types of CDs, the longstanding exposure of CDs *in vivo* and the decaying process of CDs were not studied in detail. In this study, two carbon quantum dots with high QYs, HCDs and SCDs, were prepared from the protein-rich hair and pig skin by microwave assisted carbonization treatment. As a comparison, CCDs were synthesized from citric acid and urea through microwave irradiation method. For HCDs and SCDs, the carbonization process at 300 °C under a nitrogen atmosphere decomposed the macromolecular chain of protein, drove away the small molecule in hair and pig skin and finally retained the main composition of carbon. The microwave treatment for post-carbonized HCDs and SCDs benefited the passivation of surface and the formation of surface defect, which can enhance the fluorescence efficiency. The characterization results confirmed that three CDs almost had the same chemical structures with massive carbonyl as well as a small amount of amino and hydroxyl groups, which suggested the presence of carboxylic acid and amino/oxygen-containing functional groups on the surface of CDs. The existence of those functional groups might enhance the photoluminescence, as well as promote the excellent dispersion of CDs and the combination ability with the detection targets in aqueous systems. It was reported that nitrogen, phosphorus and sulfur could provide a new kind of surface state to enhance the fluorescence intensity and QYs, and tune the intrinsic properties of CDs^15^,^21^,^46^. The SCDs and HCDs were prepared from protein-rich skin and hair that contained microelements, like phosphorus in phospholipid and sulfur in amino acid, which might enhance the photoluminescence properties of CDs. Characterization results demonstrated that three CDs almost had the same chemical properties, and nitrogen content in three CDs was HCDs > CCDs > SCDs. However, the QYs of HCDs and SCDs were much higher than CCDs. Therefore, we inferred that it was the synergistic effect of the nitrogen contents and the raw protein-rich precursors that caused the higher QYs of SCDs and HCDs. In addition, the luminescence characterization of three CDs showed the CDs held excitation-dependent photoluminescence behaviors, which was deemed to be in connection with the surface defects, the small size of CDs and multicolor imaging with CDs as probes.

*In vivo* investigation indicated that SCDs and HCDs possessed hypotoxicity, high biocompatibility and pretty photoluminescence imaging in zebrafish, while for CCDs, the fluorescence primarily concentrated on the surface of chorion and there was almost no obvious fluorescence observed in the embryos. Previous reports showed that zebrafish chorion had nanoscale pores of approximately 0.17 μm^2^,^47^, larger than the size of CCDs (ca. 3.35 nm). Therefore, in theory, the CCDs can penetrate into embryo through the pores of the chorion, and then diffuse in the embryos, but in experimental practice, the opposite conclusion was obtained. We suppose that it is possibly related with the binding interactions of chemical composition of CCDs and the chorion of embryos, which prevent CCDs from entering into the embryos across chorion. Those results indicated that different CDs had different affinity to chorion. The longtime detection of CDs in zebrafish demonstrated that HCDs and SCDs were photostable *in vivo* for a long time and the decaying of CDs in zebrafish might contribute to exclusion of the digestive system for xenobiotics. It has been confirmed that ATP, NADH and Fe^3+^ ions had quenching effects on CDs. NADH is an important coenzyme involved in metabolic processes, which stimulates the energy production in every cell of living organisms^15^. ATP is the essential fuel and present in every cell of organism, and the Fe^3+^ ions are the cofactor of numerous enzymes and the significant component of enzyme-catalyzed reaction. Hence, the inner-body metabolite or substance also might promote the decay of CDs in zebrafish. Thus, the decay process of CDs *in vivo* may come from the synergistic effect of the metabolism process and quenching of internal substances. Meanwhile, the results indicated that three CDs could be used as highly sensitive probes for Fe^3+^ ions, ATP and NADH detection. Our results demonstrated that the as-synthesized CDs are suitable as bioprobes for long-term observation and sensor, as well as provides the theoretical basis that fluorescence probes used for different purposes should be selected not only by considering its optical properties, but also chemical properties.

By comparing with the as-synthesized three CDs in our study and the reported CDs in previous literatures, we first reported the skin derived SCDs as high biocompatibility bioprobes in zebrafish and systemically explored the different imaging mechanism of three CDs. Moreover, it was the first time to study the decay process *in vivo* as long as 15 d fluorescence imaging, and the possible decay mechanisms were also elucidated. For HCDs, although they had been reported^48^, the QYs in our study are much higher because of improved preparation process with adding the microwave treatment for post-carbonized HCDs, which benefited the passivation of surface and the formation of surface defect, resulted in the enhancement the fluorescence efficiency. For CCDs, it was reported that they were water-soluble and were applied as fluorescent ink^49^. Here, we first applied them as bioprobes *in vivo*, and confirmed that fluorescence probes should consider more, before they were used for different purposes.

In summary, HCDs and SCDs were successfully prepared by facile and economic microwave-assisted carbonization approach. The as-prepared HCDs and SCDs exhibited multicolorful fluorescence and excitation-dependent behavior with photoluminescence quantum yield as high as 86.06% (HCDs) and 51.35% (SCDs), which far surpass the QYs value of CCDs (19.73%) with almost similar chemical properties to HCDs and SCDs. The HCDs and SCDs can be applied as fluorescence bioprobes *in vivo* for detecting of special substance, because of their outstanding biocompatibility, pretty photostability, special affinity, hypotoxicity, which were confirmed by zebrafish model. The longtime tracing for CDs in zebrafish as well as the quenching testing of ATP, NADH and Fe^3+^ ions for CDs demonstrated that the decaying assay of CDs in zebrafish might be caused by the synergistic action of the metabolism process by digestive system and quenching effect of internal metabolite and substance. To our knowledge, this is the first time to report SCDs and HCDs with such high QYs, *in vivo* comparative studies of HCDs, SCDs and CCDs as bioprobes, and explore their mechanism of photostability and decay in zebrafish.

## Methods

### Ethics Statement

All animal experiments were performed in accordance with the Guiding Principles for the Care and Use of Laboratory Animals and were approved by the Animal Care Committee of Chongqing Institute of Green and Intelligent Technology, Chinese Academy of Sciences (Approval ID: ZKCQY0112).

### Materials and Reagents

Human hair collected from barbershop was washed using deionized water and ethanol, and then dried for further use. Pig skin was bought in common charcuterie. NaOH and absolute alcohol were purchased from Sinopharm Chemical Reagent Co., Ltd. The citric acid monohydrate (99.5%), urea (molecular biology grade) and quinine sulphate were purchased from Sangon Biotech (Shanghai Co., Ltd.). Nicotinamide adenine dinucleotide reduced disodium salt hydrate (NADH) and adenosine 5′-triphosphate disodium salt (ATP) were obtained from Thermo Scientific (MMAS, USA). The iron (Ш) chloride hexahydrate was purchased from Chengdu kelon chemical reagent factory (China, Chengdu). The other chemicals were of analytical grade.

### Preparations of HCDs, SCDs and CCDs

Firstly, hair was cut to small fragments (about 1 mm to 5 mm), and then thermally carbonized at 300 °C for 2 h at a heating rate of 1 °C min^−1^ under a nitrogen atmosphere. After cooling down to room temperature, the dark brown product was ground to fine powders. Then 0.1 g powder was dispersed in 10 mL deionized water and sonicated for 30 min to form a brown suspension. Subsequently, the brown aqueous suspension was heated by microwave irradiation (ER-761MD, 400 W) for 4 min to obtain CDs. Finally, the suspension was centrifuged at 12,000 rpm for 25 min at room temperature to remove the large size non-fluorescent deposits. The upper suspension was dried in the lyophilizer (Virtis BT4KXL, USA) to obtain the strong blue fluorescent CDs. The as-prepared product was referred to as HCDs, which was dried and collected for further characterization and usage.

For SCDs, the frozen pig skin was firstly defrosted at ambient temperature, and then the pig skin was cut into granular structure with a knife and soaked in 3 g/L NaOH solution. The solution was treated by ultrasonic cell crusher (3 s with interval of 2 s) for 90 min to degrease. Next, the solution was washed to neutral and dried in the oven. After that, the carbonization, microwave and post processing were conducted according to the above procedure used for HCDs. The as-prepared pig skin product was referred to as SCDs.

The CCDs were prepared by one step microwave irradiation method. 3 g of citric acid monohydrate (99.5%), 3 g urea (molecular biology grade), and 10 mL of deionized water were added into the lining of a Teflon-lined autoclave vessel (30 mL). Then the lining sealed in the vessel was heated by microwave irradiation (ER-761MD, 600 W) for 6 min. After naturally cooled down in fuming cupboard to ambient temperature, the obtained dark brown solid was ground to fine powders and then dissolved in 10 mL of deionized water. Finally, the brown aqueous suspension was centrifuged at 12,000 rpm for 25 min to remove the large size non-fluorescent deposit. After dried the upper suspension in the lyophilizer (Virtis BT4KXL, USA), the strong yellow fluorescent CDs was obtained, which was referred to as CCDs.

### Characterization of CDs

The photoluminescence properties of CDs were measured by the FL-4500 fluorescence spectrometer (Hitachi, Japan). The size distribution and morphologies of CDs nanoparticles were examined by the field-emission transmission electron microscope (TEM, Tecnai G2 F20) at an accelerating voltage of 200 kV. The crystalline structures of three CDs were characterized by X-ray powder diffraction (XRD, BRUKER D8 ADVANCE), using Cu Ka (k = 1.5405 Å) as the incident radiation. The UV-visible absorption spectra was recorded by the UV spectrophotometer (Lambda35, CASCQTS-B0001, China). FTIR spectroscopy was recorded on KBr pellets with a TENSOR 27 FTIR spectrometer (Bruker) with resolution of 2 cm^−1^, and the X-ray photoelectron spectra (XPS) were performed on a PHI-5400 (USA) K-Alpha electron energy spectrometer with a monochromated Al Kα X-ray source (hʋ = 1,486.6 eV).

### Husbandry of zebrafish

Wild-type 3-month-old adult zebrafish were raised according to standard husbandry protocols^50^, with a 14 h light/10 h dark cycle. The zebrafish were fed twice daily with decapsulated freshly hatched brine shrimps (Brine Shrimp Direct, USA). One male and two female zebrafish were transferred to a breeding tank at night before the start of breeding. The next morning, sufficient embryos were collected within 1 h from the breeding tanks after the light was switched on, and then washed the embryos using the deionized water. At 4 hours post-fertilization (hpf), the embryos were examined under a dissecting light microscope (SZ760) and normally developed embryos were selected for further experiments.

### Biocompatibility assay and fluorescence imaging of CDs in zebrafish

Different concentration of CDs were confected using deionized (DI) water as solvent. Then 50 embryos at 4 hpf were placed into each well of six-well plates with 8 ml different concentration of CDs (as descripted in Fig. S9), and DI water was used as control without CDs. After the embryos were exposed to different concentrations of CDs for a certain time, the CDs were replaced with DI water and zebrafish embryos were raised until the stated time. Three replicate trials were conducted for each group. Microscopic images were taken by the Nikon SMZ18 NIS-Elements BR Stereo Fluorescence Microscope under different excitation spectra, after washing the embryos for 6 times.

The 10-day-old zebrafish were investigated by the same method above, the zebrafish embryos were normally cultured for 10 days. Then 20 healthy zebrafish larvae were selected and exposed to different CDs for 2 days. The effect of CDs on larvae was traced by taking microscopic images. Three replicate trials were conducted for each group.

### Microinjection of CDs into embryos

The microinjection of CDs to zebrafish embryos was carried out on the Eppendorf electric microinjector (FemtoJet 4i) and SZ760 series stereo microscope (Chongqing Optec Instrument Co., Ltd., China). 2 mg/ml CDs solution was prepared in sterile deionized water and used the capillary tube as an injection needle after pulling at the heating temperature of 600 °C (pull pressure was 55 pa, pull time was 10 s). And the tip of the needle was carved around 0.05 mm. 100 embryos at 0.5 hpf were placed in the agarose injection slot, then set the injection pressure to 30 psi, and the injection time interval to 30 msec. The magnification of the injection microscope was 2×10, and the injection volume was about 1~2 nl for each embryo.

### The quenching effects of ATP, NADH and Fe^3+^ ions on CDs

The quenching effects of ATP, NADH and Fe^3+^ ions were detected by the microplate reader. In every well of 96-well plates, 20 μL of CDs and 180 μL of different concentration of ATP, NADH and Fe^3+^ ions (dissolved in 0.1 M PBS with PH 7.4) were added respectively. After an interval of 10 min with gently shaking, the fluorescence spectra of the samples were recorded at the emission wavelengths (λ_em_ = 360 nm) under the optimal excitation wavelengths (λ_ex_ = 340 nm), respectively. The quenching effect was tested by the microplate reader (Infinite M200pro, USA), and calculated by the percentage of the difference values of photoluminescence intensity between the control and treated samples.

### QYs measurements

The QYs detection test of three CDs were performed by using the quinine sulfate (0.1 M H_2_SO_4_ as solvent; QYs = 0.54) as standard, and the QYs were calculated according by the following equation (1)^17^.


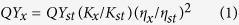


where QYs are the quantum yields, K is the slope of linear fitted by a series of photoluminescence intensity data (λex = 340 nm, λem = 360 nm) obtained at the corresponding absorbance values (less than 0.1 at 340 nm). η is the refractive index (1.33 for DI water and 0.1 M H_2_SO_4_ aqueous solution). The subscript st refers to the qunine sulfate standard and x refers to the CDs samples. For these aqueous solutions, the *η*_*x/*_*η*_*st*_ = 1. A series of concentrations with absorbance values less than 0.1 for the references and the CDs samples were measured to obtain the slopes.

## Additional Information

**How to cite this article**: Zhang, J.-H. *et al.* *In vivo* characterization of hair and skin derived carbon quantum dots with high quantum yield as long-term bioprobes in zebrafish. *Sci. Rep.* **6**, 37860; doi: 10.1038/srep37860 (2016).

**Publisher's note:** Springer Nature remains neutral with regard to jurisdictional claims in published maps and institutional affiliations.

## Supplementary Material

Supplementary Information

## Figures and Tables

**Table 1:** Element contents and the percentage of chemical bond of the CDs.

sample	Element content (%)				Percentage of Chemical bond (%)					
	C1s	N1s	O1s	Other	C1s			N1s		
					C-N	C-O/C=O	C-C	N-O	-NH_2_/-NH	N-C
HCDs	74.66%	9.66%	14.75%	0.93%	29.06%	25.28%	45.66%	35.70%	35.59%	28.71%
SCDs	82.93%	2.43%	13.52%	1.12%	5.31%	6.75%	87.95%	—	16.78%	83.22%
CCDs	73.60%	6.87%	19.09%	0.44%	10.56%	18.50%	70.93%	46.05%	37.70%	16.25%
