# Supplementary material for: In vivo characterization of hair and skin derived carbon quantum dots with high quantum yield as long-term bioprobes in zebrafish
Source: Sci Rep. 2016 Nov 25;6:37860. doi: 10.1038/srep37860 (PMC5122948; doi:10.1038/srep37860)
Supplement: Supplementary Information [file srep37860-s1.pdf]

Supporting Information

In vivo characterization of hair and skin derived carbon quantum dots with high quantum yield as long-term bioprobes in zebrafish

Running Head: *In vivo* comparative studies of carbon quantum dots

Jing-Hui Zhang[a,b]¶, Aping Niu[b]¶, Jing Li[a], Jian-Wei Fu[a], Qun Xu[a]*, De-Sheng Pei[b]*

[a]College of Materials Science and Engineering, Zhengzhou University, Zhengzhou 450052, China.

[b]Chongqing Institute of Green and Intelligent Technology, Chinese Academy of Sciences, Chongqing 400714, China.

¶These authors contributed equally to this work.

*Corresponding authors. E-mails: qunxu@zzu.edu.cn (Q.X), deshengpei@gmail.com (D.S.P) and peids@cigit.ac.cn (D.S.P)

Table S1. The QYs of SCDs, HCDs and CCDs by using quinine sulfate as reference*.

	SCDs	HCDs	CCDs
QY (%)	51.35	86.06	19.73

*The QYs are representative of three independent experiments performed in triplicate.

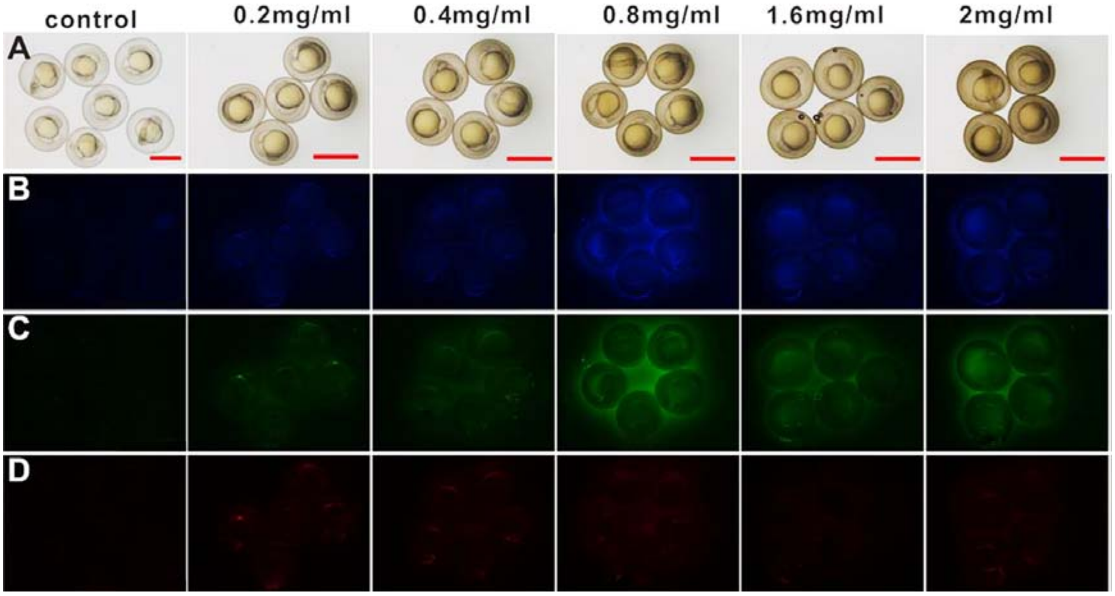

Fig. S1. Biocompatibility detection of CCDs in zebrafish embryos at 24 hpf. Fluorescent microscopic images were captured in brightfield (A) and fluorescent field (B blue; C green; D red) after embryos exposure to different concentration of CCDs for 20 h. Scale bars, 1000 μm.

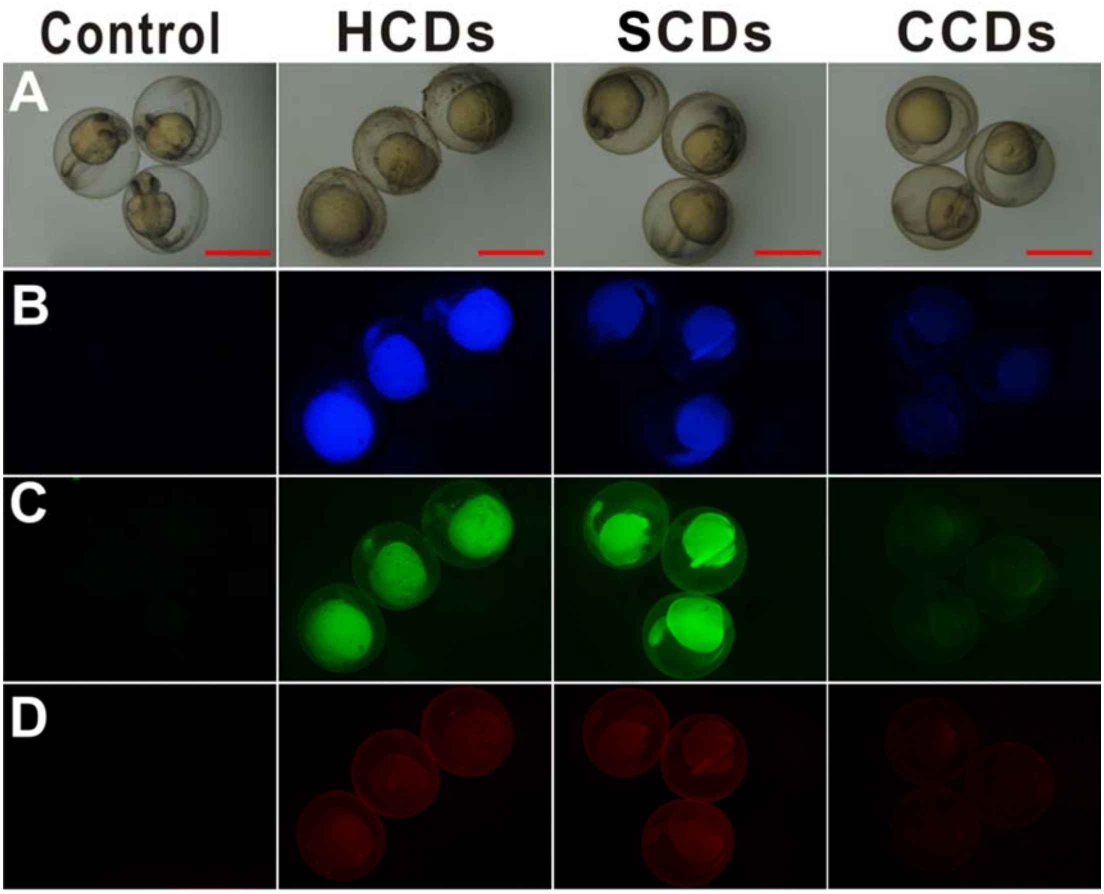

Fig. S2. Biocompatibility detection of three CDs in zebrafish embryos at 48 hpf. Fluorescent microscopic images were captured in bright field (A) and fluorescent field (B blue; C green; D red) after embryos exposure to 0.4 mg/ml different CDs for 44 h. Scale bars, 1000 μm.

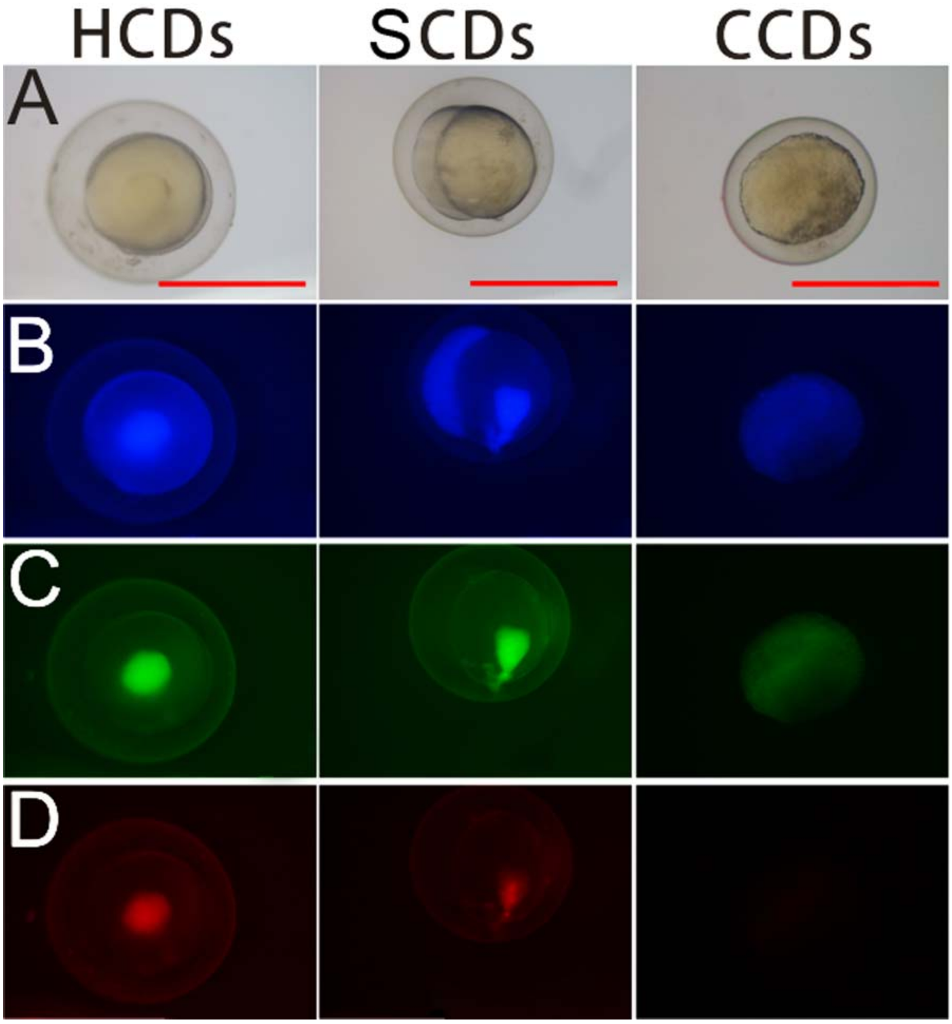

Fig. S3. Biocompatibility detection of three injected CDs in zebrafish embryos during the cleavage period. Fluorescent microscopic images were captured in bright field (A) and fluorescent field (B blue; C green; D red) after embryos injected different CDs for 30 min. Scale bars, 1000 μm.

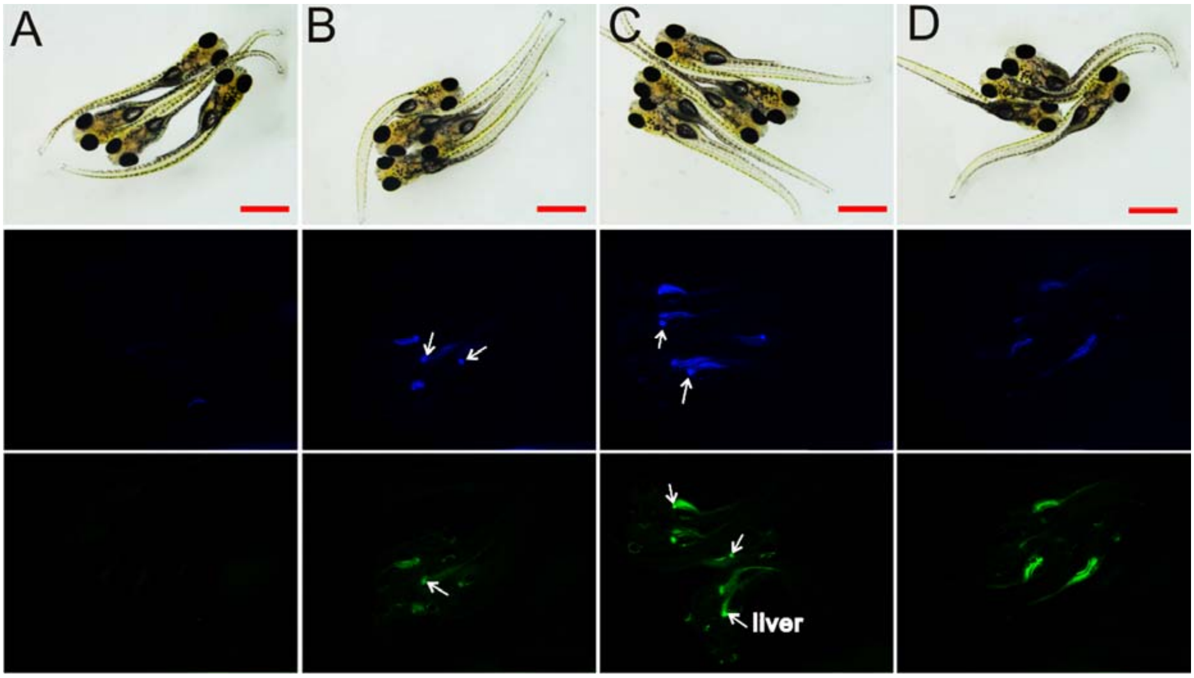

Fig. S4. Biocompatibility detection of CDs in zebrafish embryos at 10 dpf. Fluorescent microscopic images were captured in bright field (upper row) and fluorescent field (middle row and lower row) after embryos exposure to CDs for 2 d. (A) control; (B) HCDs; (C) SCDs; (D) CCDs. Scale bars, 1000 μm.

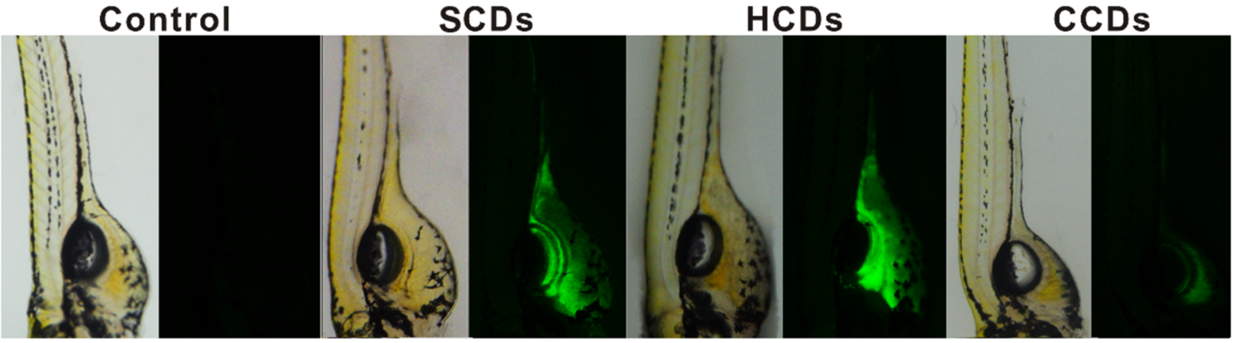

Fig. S5. The fluorescence distribution of CDs in zebrafish larvae at 5 dpf. The fluorescent images were captured in brightfield (left) and fluorescent field (right) after embryos exposure to CDs solutions for 116 h.

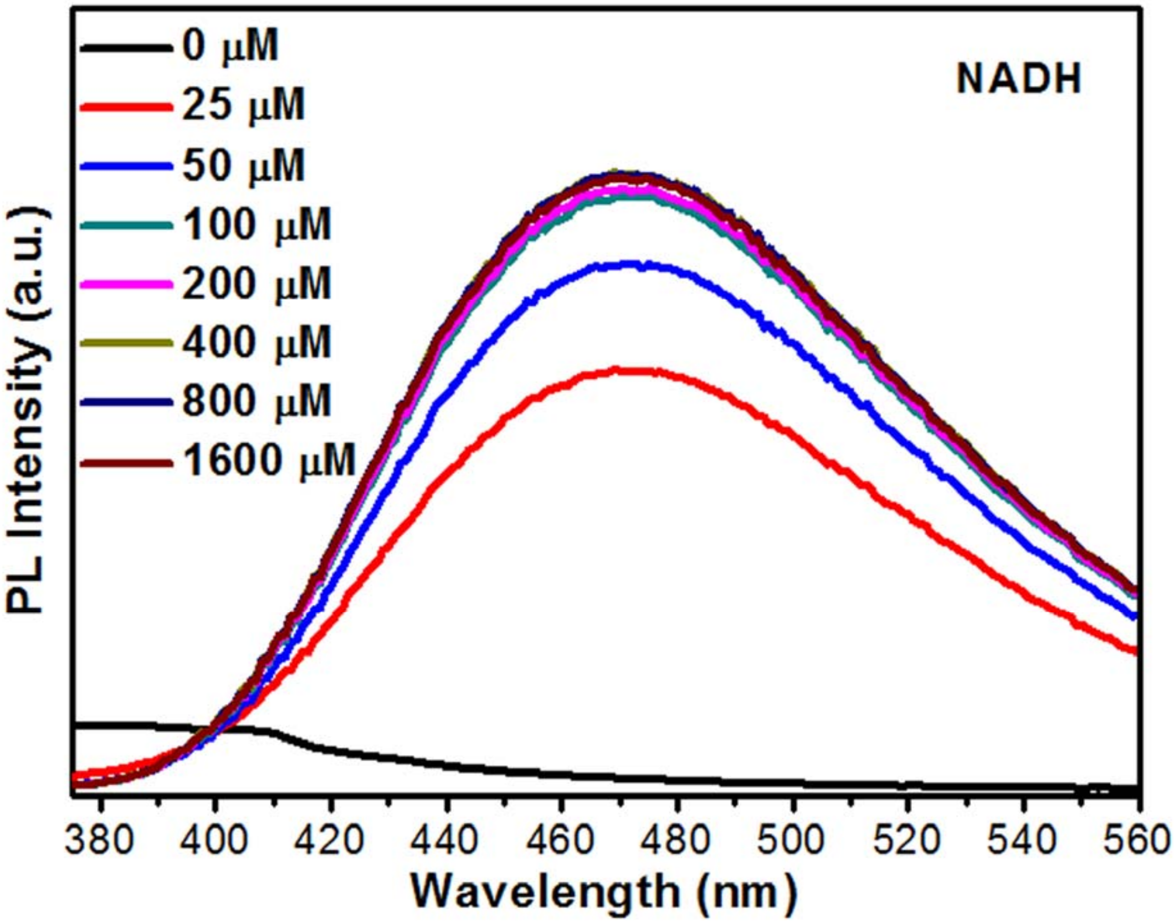

Fig. S6. PL emission spectra of different concentrations of NADH in PBS buffer.

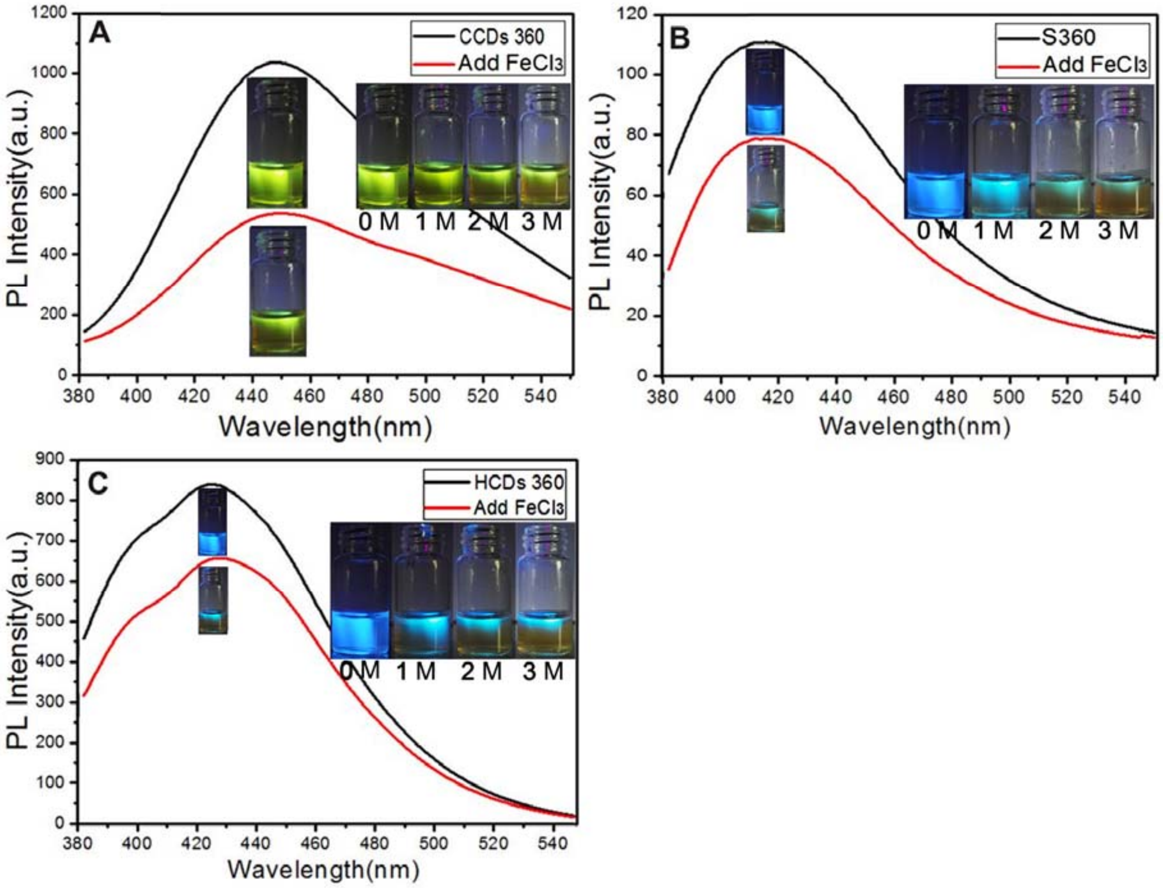

Fig. S7. Trend analyses of PL intensity. After added the same amount of Fe^{3+} ions solution at the excitation wavelength of 360 nm, PL intensity of CDs decreased. Inset: the fluorescent images of CDs in deionized water solution under the UV light with adding increasing amount of Fe^{3+} ions solution (0-3 M $FeCl_3$). A: HCDs; B: SCDs; C: CCDs.

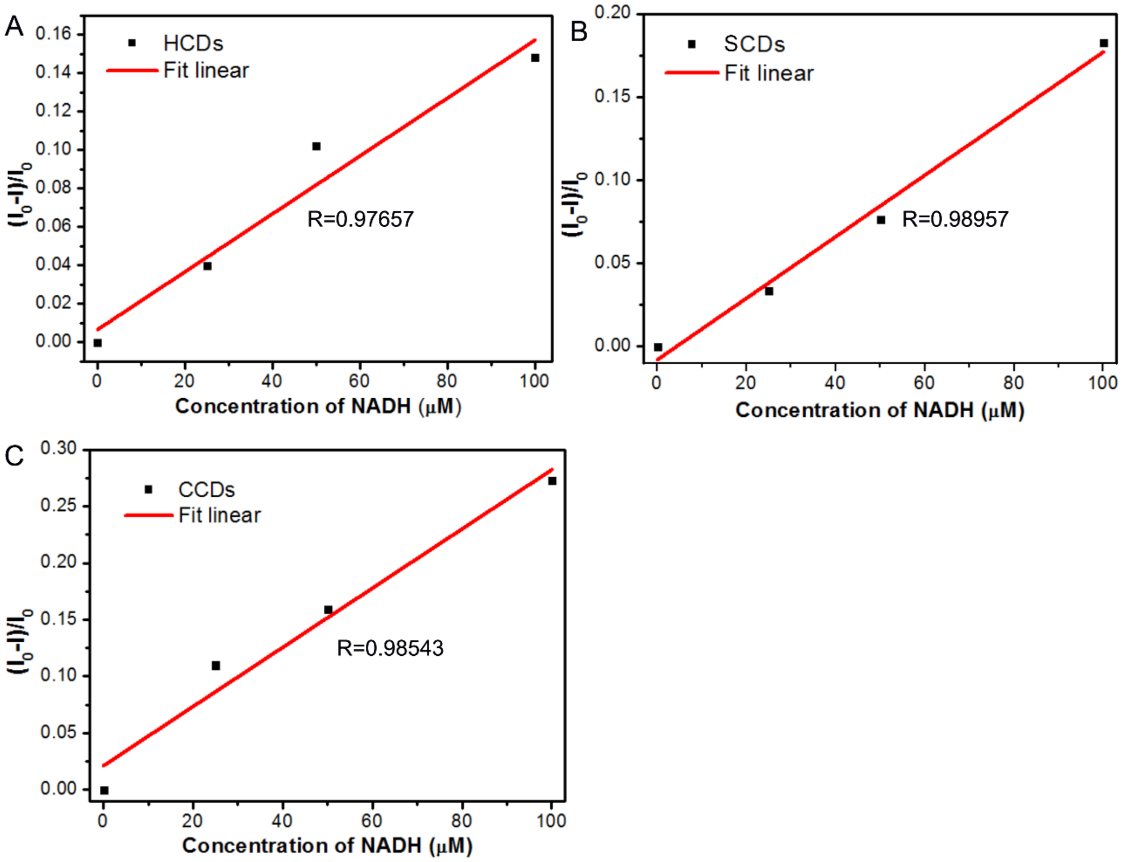

Fig. S8. The linear region of the relationship between $(I_0 - I)/I_0$ and NADH from 0 to 100 μM. I_0 and I are the FL intensities of CDs at 340 nm excitation in the presence and absence of NADH, R is the linear correlation coefficient. A: HCDs; B: SCDs; C: CCDs.

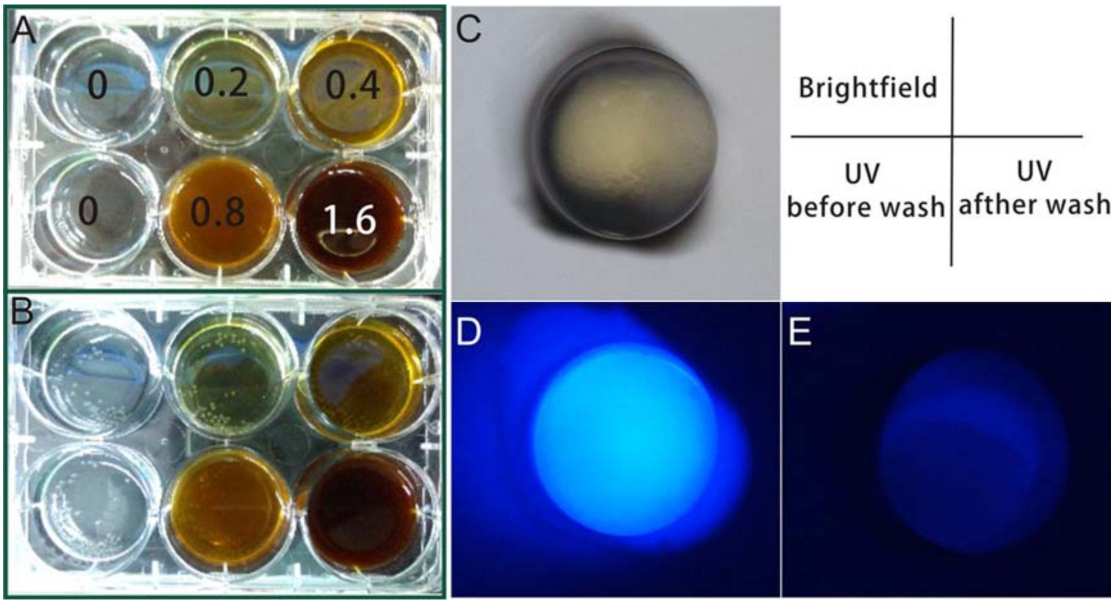

Fig. S9. Schematic diagram of CDs exposure to zebrafish. (A) Preparation of different concentrations of CDs; (B) Exposure zebrafish embryos to CDs; (C-E) The fluorescent microscopic images of zebrafish embryos in brightfield and fluorescent field under UV light before and after washed. It indicates that the washing process is essential before taking fluorescent images.
